# Recent advances of Cas12a applications in bacteria

**DOI:** 10.1007/s00253-021-11243-9

**Published:** 2021-03-23

**Authors:** Meliawati Meliawati, Christoph Schilling, Jochen Schmid

**Affiliations:** 1 Institute for Molecular Microbiology and Biotechnology, University of Münster, Corrensstrasse 3, 48149 Münster, Germany; 2 Chair of Chemistry of Biogenic Resources, Campus for Biotechnology and Sustainability, Technical University of Munich, Schulgasse 16, 94315 Straubing, Germany

**Keywords:** CRISPR-Cas12a, Genome editing, Transcriptional perturbation, Multiplex gene regulation

## Abstract

**Abstract:**

Clustered regularly interspaced short palindromic repeats (CRISPR)-mediated genome engineering and related technologies have revolutionized biotechnology over the last decade by enhancing the efficiency of sophisticated biological systems. Cas12a (Cpf1) is an RNA-guided endonuclease associated to the CRISPR adaptive immune system found in many prokaryotes. Contrary to its more prominent counterpart Cas9, Cas12a recognizes A/T rich DNA sequences and is able to process its corresponding guide RNA directly, rendering it a versatile tool for multiplex genome editing efforts and other applications in biotechnology. While Cas12a has been extensively used in eukaryotic cell systems, microbial applications are still limited. In this review, we highlight the mechanistic and functional differences between Cas12a and Cas9 and focus on recent advances of applications using Cas12a in bacterial hosts. Furthermore, we discuss advantages as well as current challenges and give a future outlook for this promising alternative CRISPR-Cas system for bacterial genome editing and beyond.

**Key points:**

• *Cas12a is a powerful tool for genome engineering and transcriptional perturbation*

• *Cas12a causes less toxic side effects in bacteria than Cas9*

• *Self-processing of crRNA arrays facilitates multiplexing approaches*

## Introduction

In basic research and industrial biotechnology, genetic engineering is essential for genomic and metabolic manipulation to direct microorganisms towards production of specific valuable products. For this, accessibility of the genome in combination with a highly efficient molecular tool are essential factors for the generation of versatile and robust chassis organisms. In the past decades, vast research has been directed on the development of techniques, which improve genome editing and gene regulation in various microorganisms. The breakthrough by the discovery of the CRISPR-Cas technology has shed light on the adaptive immune system of prokaryotes, and since then opened up tremendous opportunities for targeted genetic engineering approaches in pro- and eukaryotes (Jinek et al. 2012). CRISPR (clustered regularly interspaced short palindromic repeats) is an RNA-guided adaptive defense mechanism in bacteria and archaea that protects them against invasion of viruses and foreign genetic materials (Barrangou et al. 2007; Jinek et al. 2012; Makarova et al. 2020). Native CRISPR-Cas systems have been detected in more than 85% of analyzed archaeal genomes and 40% of bacterial genomes. Today, updated classification of the CRISPR-Cas systems comprises 2 classes, 6 types, and 33 subtypes. Among all CRISPR systems that have been discovered yet, Cas9 from *Streptococcus pyogenes* is still by far the most studied and well characterized. Based on the architecture of the genomic loci, it is classified as a Class 2 type II-A CRISPR system for its single, large effector Cas9 protein (Makarova et al. 2020). Throughout the years, many studies have been investigating the activity and potential applications of Cas9 for genetic engineering purposes (Adli 2018; Pickar-Oliver and Gersbach 2019).

The more recently discovered Cas12a, which belongs to the Class 2 type V-A CRISPR system, has garnered a lot of attention for its attractive features and potential applications (Zetsche et al. 2015). Despite coming from the same class and postulated to have evolved similarly, Cas9 and Cas12a hold some intrinsic differences which may distinguish their practical applications (Mohanraju et al. 2016; Koonin et al. 2017; Swarts and Jinek 2018). First, both of them recognize different protospacer adjacent motifs (PAM) and have different mechanisms to perform the double strand break. Cas9 recognizes the PAM sequence 5′-NGG, cleaves at proximal position of the PAM, and generates blunt end double-strand breaks (Deltcheva et al. 2011; Jinek et al. 2012). In contrast, Cas12a recognizes T-rich sequence 5′-TTTV and cleaves at distal position of the PAM, generating a staggered double strand break (Zetsche et al. 2015; Kim et al. 2017; Swiat et al. 2017). Second, looking at the domain architectures, Cas9 has two nuclease domains (RuvC and HNH) which each cleaves one strand of the double-strand DNA (dsDNA) (Gasiunas et al. 2012). Contrarily, Cas12a has only a RuvC-like domain and lacks the HNH domain, but is still able to generate a dsDNA break (Zetsche et al. 2015). Third, both types use different crRNA as well as processing mechanism to generate mature crRNA. In the case of Cas9, it needs both tracrRNA and crRNA to allow Cas9 binding to the target sequence. As Cas9 does not possess RNase activity, maturation of crRNA is dependent on the activity of the endoribonuclease RNase III (Deltcheva et al. 2011). In contrast, Cas12a only needs crRNA and does not require the additional tracrRNA. Due to the additional RNase activity, Cas12a can process maturation of the crRNA arrays itself and is therefore independent of other RNase activity (Zetsche et al. 2015; Fonfara et al. 2016). This feature makes Cas12a superior for multiplexing of different targets (Table 1).

**Table 1:** Distinct characteristics of Cas9 and Cas12a

Properties	Cas9	Cas12a
CRISPR system classification	Class 2, type II-A	Class 2, type V-A
Commonly used origin	*Streptococcus pyogenes*	*Francisella novicida* *Acidaminococcus* sp*.* *Lachnospiraceae bacterium* *Moraxella bovoculi*
Nuclease domain	HNH and RuvC	RuvC
CRISPR-RNA	crRNA and tracrRNA	crRNA
PAM site (5′-3′); position relative to the spacer	NGG; immediate downstream of the spacer	TTTV; immediate upstream of the spacer
Cutting style	Blunt end	Staggered end
RNase activity	No	Yes
Multiplexing	+*	+++

*While multiplex approaches have been demonstrated using Cas9, several undesired side effects have been observed (McCarty et al. 2020)

Recently, Cas12a has emerged as a reliable genetic tool and attractive alternative to Cas9 (Paul and Montoya 2020). Many studies have investigated its activity in eukaryotes including plants and human cells. However, application of Cas12a in prokaryotes is still limited and it needs more investigations to explore its full potential. In this minireview, we will focus on the current different applications of Cas12a in bacteria. We will discuss challenges and obstacles and give an outlook on what can be expected for future utilization of Cas12a as a robust prokaryotic genetic engineering tool.

## CRISPR-Cas12a as an attractive system for genetic engineering

Also known as Cpf1, Cas12a was initially detected in the genome of *Francisella* and *Prevotella* strains (Schunder et al. 2013; Zetsche et al. 2015). Its activity was first demonstrated in *Escherichia coli* where its facilitation of DNA interference was shown (Zetsche et al. 2015). Since then, multiple studies have exploited the potential of Cas12a homologs from *Francisella novicida* ATCC 15482 (FnCas12a), *Acidaminococcus* sp. BV3L6 (AsCas12a), *Lachnospiraceae bacterium* ND2006 (LbCas12a), and *Moraxella bovoculi* AAX11_00205 (MbCas12a). Thus far, the Cas12a orthologs are shown to be able to mediate genome editing in human cells (Zetsche et al. 2015; Tóth et al. 2018). In recent years, Cas12a has also been employed for genome editing and gene regulation in bacteria, although the information is very limited compared to its application in eukaryotes (Yao et al. 2018; Adiego-Pérez et al. 2019; Liu et al. 2020). Among the three variants, FnCas12a has been used the most to facilitate genetic engineering in bacteria (Table 2). While some studies reported the activity of LbCas12a in yeast, higher plants, and mammalian cells (Zetsche et al. 2015; Verwaal et al. 2018; Bernabé-Orts et al. 2019; Liu et al. 2019), to the best of our knowledge, it has not been tested in prokaryotes.

**Table 2:** Overview of Cas12a applications for gene editing and regulation in bacteria

Cas12a variant	Organism	Bacterial class	Multiplexing; number of target genes	Purpose	Reference
FnCas12a	*Rhodobacter capsulatus* ATCC BAA-309**	α-proteobacteria	No	Knock-in, knock-out, point mutation	(Zhang and Yuan 2020)
FnCas12a	*Zymomonas mobilis* ATCC 31821	α-proteobacteria	No	Point mutation, gene deletion, gene replacement	(Shen et al. 2019)
FnCas12a	*Escherichia coli* ATCC 700926	γ-proteobacteria	No	Point mutation, gene replacement	(Yan et al. 2017)
FnCas12a	*Escherichia coli* ATCC 700926	γ-proteobacteria	Yes; 3	Multiplex gene integration	(Ao et al. 2018)
dAsCas12a	*Escherichia coli* PTA-5105	γ-proteobacteria	Yes; 4	Multiplex gene repression	(Zhang et al. 2017)
FnCas12a	*Yersinia pestis* KIM6+	γ-proteobacteria	No	Point mutation, gene replacement	(Yan et al. 2017)
FnCas12a	*Halomonas bluephagenesis* TD01	γ-proteobacteria	No	Point mutation, gene deletion	(Ao et al. 2018)
FnCas12a	*Pseudomonas putida* ATCC 47054	γ-proteobacteria	No	Gene deletion	(Sun et al. 2018b)
FnCas12a	*Synechococcus* sp. UTEX 2973*	Cyanophyceae	No	Knock-in, knock-out, point mutation	(Ungerer and Pakrasi 2016)
FnCas12a	*Synechocystis* sp. ATCC 27184	Cyanophyceae	No	Knock-in, knock-out, point mutation	(Ungerer and Pakrasi 2016)
FnCas12a	*Anabaena* sp. ATCC 27893	Cyanophyceae	No	Knock-in, knock-out, point mutation	(Ungerer and Pakrasi 2016)
FnCas12a	*Mycobacterium smegmatis* ATCC 700084*	Actinobacteria	No	Point mutations, gene disruption, gene replacement	(Yan et al. 2017; Sun et al. 2018a)
FnCas12a	*Corynebacterium glutamicum* ATCC 13032*	Actinobacteria	No	Point mutations, gene deletion, gene insertion	(Jiang et al. 2017; Krumbach et al. 2019; Zhang et al. 2019; Zhang et al. 2020)
FnCas12a	*Streptomyces coelicolor* ATCC BAA-471*	Actinobacteria	Yes; 2	Multiple gene deletion	(Li et al. 2018a)
dFnCas12a	*Streptomyces coelicolor* ATCC BAA-471*	Actinobacteria	Yes; 3	Multiplex gene repression	(Li et al. 2018a)
FnCas12a	*Streptomyces hygroscopicus* SIPI-KF**	Actinobacteria	No	Gene deletion	(Li et al. 2018a)
FnCas12a	*Amycolatopsis mediterranei* U32**	Actinobacteria	No	Gene deletion	(Zhou et al. 2020)
AsCas12a	*Clostridium difficile* ATTC BAA-1382	Clostridia	Yes; 2	Multiplex gene deletion	(Hong et al. 2018)
AsCas12a	*Clostridium beijerinckii* NCIMB8052	Clostridia	No	Gene deletion	(Zhang et al. 2018)
FnCas12a	*Bacillus subtilis* DSM 402	Bacilli	Yes; 2	Multiplex gene deletion	(Wu et al. 2020)
dFnCas12a	*Bacillus subtilis* DSM 402	Bacilli	Yes; 3	Multiplex gene repression, simultaneous repression-activation	(Wu et al. 2020)
dAsCas12a	*Paenibacillus polymyxa* DSM 365	Bacilli	Yes; 4	Multiplex gene repression, simultaneous repression-activation	(Schilling et al. 2020b)

*Toxicity of Cas9 has been reported in these bacteria

**No studies on Cas9-mediated genetic engineering have been reported

Since its discovery, Cas12a has arisen as a potential genetic tool and promising alternative to Cas9. One of the common drawbacks of Cas9 is its toxicity that has been reported in different bacteria (Wendt et al. 2016; Ye et al. 2020). Although the reasons are still poorly understood, it is postulated that, in some bacteria, Cas9 might bind non-specifically to the PAM even without a guide RNA (Jiang et al. 2017; Jones et al. 2017). This will subsequently interfere with gene expression and regulation throughout the genome considering the abundant presence of 5′-NGG site (Cho et al. 2018). Different studies indicate that Cas12a has a less toxic effect, which makes it highly promising for CRISPR-Cas-based genetic engineering in prokaryotes (Jiang et al. 2017). Different toxicity levels of the two Cas proteins is obvious in the cyanobacterium *Synechococcus* sp. 2973, where promoterless Cas9 is even more toxic than Cas12a expressed under control of a lac promoter (Ungerer and Pakrasi 2016).

## Cas12a-mediated genome editing

In bacteria, most common application of CRISPR-Cas system is intended for genome editing. As an RNA-guided endonuclease, similar to Cas9, Cas12a also requires crRNA containing specific spacer sequence to guide the effector module to the target region. In its native system in *Francisella novicida* U112, mature crRNA consists of 19 nts of a direct repeat followed by 23–25 nts spacer sequence. The nucleotides at position 1–5 represent the “seed” sequence which is essential in establishing stable binding between crRNA and the target strand (Zetsche et al. 2015). It is important that the seed sequence perfectly matches the target strand as mutations in this region will abolish or significantly reduce the nuclease activity of the Cas protein (Semenova et al. 2011; Swarts et al. 2017).

Once the crRNA binds to the target strand, active Cas12a will cleave the target sequence at the distal end of the protospacer (Zetsche et al. 2015), causing a dsDNA break in the chromosome. To maintain genome integrity, the organism has to repair the lethal DSB. Several repair mechanisms have been described so far, like the homology-directed repair (HDR), non-homologous end joining pathway (NHEJ), and alternative-end joining (A-EJ) (Szostak et al. 1983; Chayot et al. 2010; Lieber 2011). While NHEJ is commonly used by eukaryotes, most bacteria primarily rely on HDR to repair the DNA break (Hiom 2009). In bacteria with an active NHEJ pathway like *Mycobacterium smegmatis*, Cas12a can be utilized for gene disruption studies to investigate its function (Sun et al. 2018a). However, NHEJ-mediated repair often results in random insertions and deletions, which makes it difficult to achieve targeted genome editing. For precise genetic engineering, HDR is often employed to introduce desired modifications to bacterial chromosomes (Ran et al. 2013; Rütering et al. 2017; Schilling et al. 2020a). Utilization of CRISPR-Cas systems greatly reduce the screening effort as the double-strand break caused by the Cas-nuclease is lethal to non-edited cells.

Cas12a has been successfully tested in bacteria from different classes and ecological niches, implying the versatility of the system despite more restrictive PAM sites (Tóth et al. 2020), which results in a ~4.4-fold decreased PAM frequency compared to Cas9 in the genome of *E. coli* K12. Furthermore, in bacteria where expression of active Cas9 is toxic, e.g., in GC-rich organisms like *Corynebacterium glutamicum*, Cas12a can successfully facilitate genome editing (Zhao et al. 2020). Nevertheless, it is important to note that different bacteria strains might have different response to the introduced CRISPR-Cas system. In the case of *C. glutamicum*, it was observed that one out of three tested strains could somehow escape Cas12a cleavage (Jiang et al. 2017).

Multiple approaches have been established to make Cas12a-based genome editing more robust and fit the experimental design. In the most minimalistic setup, all-in-one plasmid systems are often used. The plasmid carries everything needed to realize the editing: Cas12a, crRNA array, and homology flanks as repair template (Jiang et al. 2017). Two plasmids system where Cas12a and crRNA array are expressed from different plasmids are also well exploited. Depending on the application, the latter is particularly beneficial when coupling CRISPR-Cas with other genetic engineering systems like recombineering (Yan et al. 2017). For this, Cas12a and recombineering genes are combined in one plasmid and used to transform the host strain first. A second plasmid containing the crRNA array is then used to co-transform the host with the oligonucleotides template. Such systems simplify the work when multiple individual targets and mutations are desired, especially in the strains with limited recombination frequencies like mycobacteria (van Kessel and Hatfull 2007). The coupled system is very convenient when aiming for iterative mutagenesis since it skips many laborious cloning steps (Jiang et al. 2017). To date, Cas12a-assisted recombineering has been successfully employed to achieve different point mutations and gene manipulations in *E. coli*, *Yersinia pestis*, *Mycobacterium smegmatis*, *C. glutamicum*, *Zymomonas mobilis*, and the halophilic bacterium *Halomonas bluephagenesis* (Jiang et al. 2017; Yan et al. 2017; Ao et al. 2018; Shen et al. 2019)*.* In *C. glutamicum*, it was reported that Cas12a-assisted RecET system realized large deletion up to 20 kb with an efficiency of 36.4% (Zhao et al. 2020), slightly higher than Cas9-RecET system with 26.9% (Wang et al. 2018).

Further developments have been made to increase Cas12a-mediated genome editing efficiency. As seen with recombineering, combining Cas12a with other genome editing tools could be beneficial, especially for systems with low efficiency. Zhang et al. (2020) demonstrated that combination of traditional SacB counterselection with CRISPR-Cas12a dramatically increased efficiency of gene insertion and deletion in *C. glutamicum* (Zhang et al. 2020). Today, Cas12a utilization has gone beyond simple proof-of-principle of genetic manipulations to directed metabolic engineering for production of high-value products such as amino acids, platform chemicals, or polysaccharides (Zhang et al. 2020; Krumbach et al. 2019; Schilling et al. 2020b). Elimination of competing pathways, release of product inhibition, and fine tuning of targeted pathways could be realized by Cas12a-assisted systems (Zhang et al. 2019, 2020; Schilling et al. 2020b).

## Cas12a-mediated gene activation and repression

**Fig. 1:**
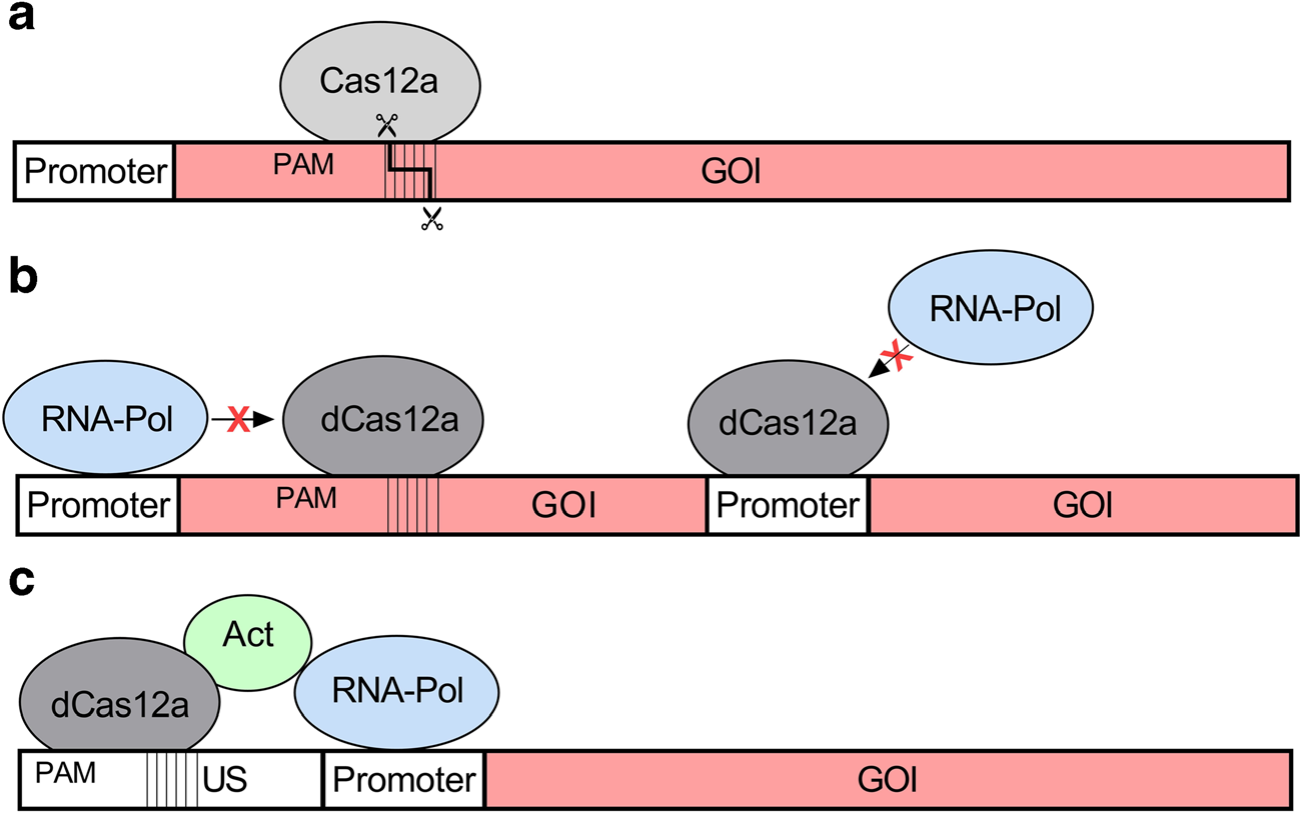
Different modes of action of CRISPR-Cas12-based applications in bacteria. **a** Staggered double-strand DNA cleavage after binding of crRNA-Cas12a effector complex to the DNA, which can be used to promote homology-directed repair or non-homologous end joining for genome editing efforts. **b** CRISPRi with catalytically inactive Cas12a variants (dCas12a) can no longer induce DNA cleavage. dCas12a either blocks elongation of transcription acting as a roadblock or prevents binding of the RNA-polymerase to the target promoter site and thereby reduces expression of a gene of interest (GOI). **c** CRISPRa uses dCas12a fused to a transcriptional activator binding to the upstream (US) region of a target promoter to facilitate the recruitment of RNA-polymerase and thereby enhances expression of a GOI

Today, the available CRISPR-Cas systems not only allow gene editing but also gene regulation by means of catalytically inactive DNase-dead Cas (dCas) variants (Fig. 1). For this, dCas can be harnessed for both gene repression by CRISPR interference (CRISPRi) and activation (CRISPRa) and therefore is appealing to tune the level of gene expression. Particularly for targeting essential genes, for which knock-outs would be lethal, knock-down via CRISPRi offers an effective solution to redirect carbon fluxes to desired products. Moreover, utilization of dCas12a can be a quick and straightforward strategy to screen for multiple target genes simultaneously since it only requires the expression of dCas12a and crRNA arrays without the need to supply a homology repair template. Different dCas12a variants have been engineered by introduction of mutations in the RuvC domain (Table 3).

**Table 3:** Different mutations needed for dCas12a generation

dCas12a variants	Mutation	Reference
dFnCas12a	D917A E1006A	(Zetsche et al. 2015)
dAsCas12a	D908A E993A	(Yamano et al. 2016)
dLbCas12a	D832A E925A	(Miao et al. 2019)
dMbCas12a	D864A	(Knott et al. 2019)

Compared to gene editing, dCas12a-mediated gene regulation in bacteria was reported less frequently although some studies demonstrated its high efficiency for gene interference (Table 2). Thus far, dCas12a has only been tested in *E. coli*, *Bacillus subtilis*, *Streptomyces coelicolor*, and *Paenibacillus polymyxa* (Zhang et al. 2017; Li et al. 2018a; Schilling et al. 2020b; Wu et al. 2020)*.* A clear strand bias of the repression efficiency by dCas12a was observed, especially when aiming for interference during transcription elongation. Different studies reported that efficiency of transcriptional perturbation significantly increases when the template strand is targeted (Zhang et al. 2017). In *S. coelicolor*, repression efficiency of dFnCas12a targeting the template strand can achieve up to 88% whereas it was much less effective when targeting the non-template strand (Li et al. 2018a). Contrarily, dCas9 has shown high preference towards the non-template strand (Tong et al. 2015). In addition, it is also observed that repression efficiency is highest when crRNAs target the site closest to the transcription start site. However, strand bias does not seem to affect dCas12a efficiency when targeting transcription initiation by blocking the promoter region (Zhang et al. 2017). Particularly for transcriptional perturbation, optimal crRNA design is essential to ensure a tight binding of the effector module. Miao et al. (2019) demonstrated that also the PAM sequence and surrounding nucleotides can highly influence the dynamic range of transcriptional perturbation (Miao et al. 2019).

Besides CRISPRi, dCas12a can also be employed for activation of gene expression by linking it to a transcription activator domain. Upon dCas12a binding to the target region, the activator domain facilitates recruitment of RNA polymerase leading to higher expression levels of the gene of interest. Gene activation facilitated by the dCas12a has been well explored in mammalian cells (Campa et al. 2019; Kleinstiver et al. 2019). However, its exploitation in bacteria is very limited and has only been demonstrated in *B. subtilis* and *P. polymyxa* (Schilling et al. 2020b; Wu et al. 2020)*.* These studies demonstrated that linking dCas12a to transcription activation domain like RemA or SoxS resulted in higher expression levels of the target genes. Contrary to eukaryotic organisms, for which CRISPRa is primarily based on chromatin rearrangements (Gilbert et al. 2013), for bacterial applications, the activator domain needs to be positioned in a precise distance to the promoter region to activate transcription (Dong et al. 2018). Currently, empirical testing of different crRNAs is required to optimize the dynamic output. However, with an increasing number of studies, it will be possible to develop clear design rule sets for different bacterial promoters to enable efficient experimental design a priori. Interestingly, both studies mentioned above also explored the potential of simultaneous activation and repression by positioning the dCas12a either adjacent to the promoter region to activate transcription initiation, or within the gene to block transcription elongation. These findings once more display the versatility of CRISPR-Cas12a systems, especially when multiple gene targeting is desired.

## Multiplex Genome Editing and Regulation

While Cas12a is of importance for bacterial strains in which Cas9 expression shows toxic effects, its simplicity for multiplex targeting remains the most attractive property of Cas12a. To realize multiplex targeting, the spacers-containing crRNAs can either be delivered individually in separate plasmids or in form of a crRNAs array. Nonetheless, it has been reported that supplying the crRNAs in one array is as efficient as supplying them individually (Ao et al. 2018). Therefore, the latter strategy is often used for its simplicity, making use of Cas12a ability to self-process the maturation of crRNAs. It is remarkable to observe that the order of crRNA generally does not affect editing and repression efficiencies (Zhang et al. 2017), although there are exceptions for some specific genes or genomic areas (Li et al. 2018a).

Despite the great potential, Cas12a-based multiplexing has only been investigated in few bacteria: *E. coli*, *B. subtilis*, *Clostridium difficile*, *S. coelicolor*, and *P. polymyxa* (Zhang et al. 2017; Ao et al. 2018; Hong et al. 2018; Li et al. 2018a; Schilling et al. 2020b; Wu et al. 2020)*.* Nevertheless, the studies demonstrated the functionality of Cas12a multiplexing with reasonably high efficiency. In bacteria, the highest degree of multiplexing that has been investigated thus far was regulation of four genes in *E. coli* and *P. polymyxa* (Zhang et al. 2017; Schilling et al. 2020b)*.* While efficiency of transcriptional perturbation is usually not heavily influenced by an increasing number of targets, efficacy of genome editing via homology-directed repair can decrease (Zhang et al. 2017; Li et al. 2018a). Multiplexing of two gene deletions in *C. difficile* resulted in an efficiency of 25% which was significantly lower than the efficiency of targeted single gene deletion (Hong et al. 2018). In *E. coli*, where single-site chromosomal integration showed an efficiency close to 100%, it dropped to 40% and 20% when two and three loci were targeted for simultaneous integrations (Ao et al. 2018). In contrast, Li et al reported 75% efficiency of simultaneous knock-out of two genes in *S. coelicolor* (Li et al. 2018a)*.*

## Optimization of Cas12a activity

There are different strategies that can be employed to achieve higher activity of Cas12a in the desired bacterial host. An important aspect is to ensure adequate expression of Cas12a. Since each organism has distinct codon usage preference (Quax et al. 2015), it is essential that the heterologously expressed Cas12a can be translated at an appropriate level. With decreasing cost of gene synthesis, nowadays, codon-optimized Cas12a is a common starting point in establishing the system especially in bacterial strains where its activity has not yet been investigated. Codon optimization is beneficial to increase the pool of mature Cas12a which could lead to higher efficiency (Ao et al. 2018). Due to its relatively low toxicity, constitutive expression of Cas12a is generally not an issue. In fact, it is preferable in some cases where inducible expression could not provide sufficient efficiency (Li et al. 2018a).

Various studies also investigated different possibilities to enhance the activity of Cas12a. It is reported that engineered AsCas12a variant with E174R/S542R/K548R mutations has twofold higher editing efficiency in human cells than the wild-type variant (Kleinstiver et al. 2019). Furthermore, several variants which recognized non-canonical PAM sites have been designed, which extend the genomic region that can be targeted by the nuclease. It has been demonstrated that AsCas12a carrying the mutations S542R/K607R and S542R/K548V/N552R shows altered PAM recognition to TYCV and TATV, respectively, with improved activities when tested in vitro and in human cells (Gao et al. 2017). When the corresponding mutations are introduced to FnCas12a, LbCas12a, and MbCas12a, the new variants are able to facilitate efficient genome editing with altered PAM recognition (Zhong et al. 2018; Tóth et al. 2020). Based on the observation on how these mutations can be applied to different Cas12a homologs and eukaryotic host organisms, it is reasonable to hypothesize that it will also be relevant for application in bacteria. Engineered Cas12a variants are substantial extensions to currently available bacterial genetic tools, increasing the efficiency of the nucleases or broadening the repertoire of possible PAM to engineer otherwise inaccessible targets or minimize off targeting effects (Kleinstiver et al. 2019). However, most engineered variants of Cas12a have been exclusively tested in eukaryotic organisms and increased efficacy in bacteria remains to be investigated.

## Future outlook

First characterized in 2015, Cas12a has emerged as a promising genetic tool and many studies have exploited its potential since then. With the rapidly growing research, there will be several improvements that we can anticipate in the upcoming years which will boost the use of Cas12a for bacterial genome engineering.

As often seen in biological systems, there exist antagonistic mechanisms to keep the balance of the natural condition. Recently, it was described that some proteins can act as natural inhibitor of Cas nucleases (Pawluk et al. 2018). Although it is rarely used in practical applications up to now, the so-called anti-CRISPR (Acr) protein represents an appealing approach for various future applications. Understanding of Acr is especially of importance when working with bacterial strains that encode endogenous CRISPR-Cas system, since many of these bacteria also encode native *acr* genes. For example, *Listeria monocytogenes* encodes *acr* for Cas9. Consequently, it severely inhibits commonly used SpCas9 (Marino et al. 2020). Since many Acr proteins inhibit only one specific subtype, it will be interesting to see if it is a feasible approach to use Acr to suppress the native CRISPR system, while at the same time introducing another type of CRISPR-Cas system to facilitate genetic engineering. Furthermore, Acr can also be used to achieve programmable CRISPR-Cas activity at a specific time to alleviate the toxicity of Cas proteins which may result in higher transformation and editing efficiencies (Marino et al. 2020).

To broaden Cas12a application, it will also be interesting to analyze its utilization as a highly efficient base editing tool in bacteria. As described for Cas9, fusing the dead or nickase variant with a cytidine deaminase protein could direct the conversion of cytosine to thymidine within a particular editing window (Komor et al. 2016; Zheng et al. 2018). Application of Cas12a for base editing thus far has only been described for mammalian cells by means of dLbCas12a-cytidine deaminase fusion protein (Li et al. 2018b), where utilization of optimized cytidine deaminases greatly improved the base editing efficiency (Chen et al. 2020). Applying the system into bacteria would be an attractive strategy to achieve C → T-targeted point mutations or pursuit mutagenesis purposes to generate various mutant strains.

Finally, we also anticipate the development of other Cas12a variants including the nickase which only induces ssDNA breaks, while still triggering the repair mechanism. The mutated variants will particularly be of interest for applications in bacteria which are deficient of the dsDNA break repair mechanism (Song et al. 2017). To our knowledge, no Cas12a nickase has been developed so far, although a preliminary study reported that the R1226A mutation of AsCas12a showed nickase activity in vitro (Yamano et al. 2016)*.* This variant will certainly be a beneficial add-on for extended applications of Cas12a.
